# Sleep, Appetite, and Obesity—What Is the Link?

**DOI:** 10.1371/journal.pmed.0010061

**Published:** 2004-12-07

**Authors:** Patricia Prinz

## Abstract

There is a well known relationship between short sleep duration and high body mass index. A new study suggests that the missing link could be the appetite regulating hormones leptin and ghrelin.

There is a well-documented relationship between short sleep duration and high body mass index (BMI). In the largest study, a survey on sleep duration and frequency of insomnia in more than 1.1 million participants, increasing BMI occurred for habitual sleep amounts below 7–8 hours [1]. A recent prospective study found an association between sleep curtailment and future weight gain [2]. The mechanism linking short sleep with weight gain is unknown, but Mignot and colleagues' study in this month's *PLoS Medicine* [3] adds to the growing evidence implicating leptin and ghrelin, the two key opposing hormones involved in appetite regulation.

## Hormones That Regulate Appetite

Leptin, a peptide hormone secreted from white adipocytes, is implicated in the regulation of food intake and energy balance. The hormone acts on the central nervous system, in particular the hypothalamus, suppressing food intake and stimulating energy expenditure. Leptin production is primarily regulated by insulin-induced changes in adipocyte metabolism—its secretion levels correlate with adipocyte mass and lipid loads.[Fig pmed-0010061-g001]

**Figure pmed-0010061-g001:**
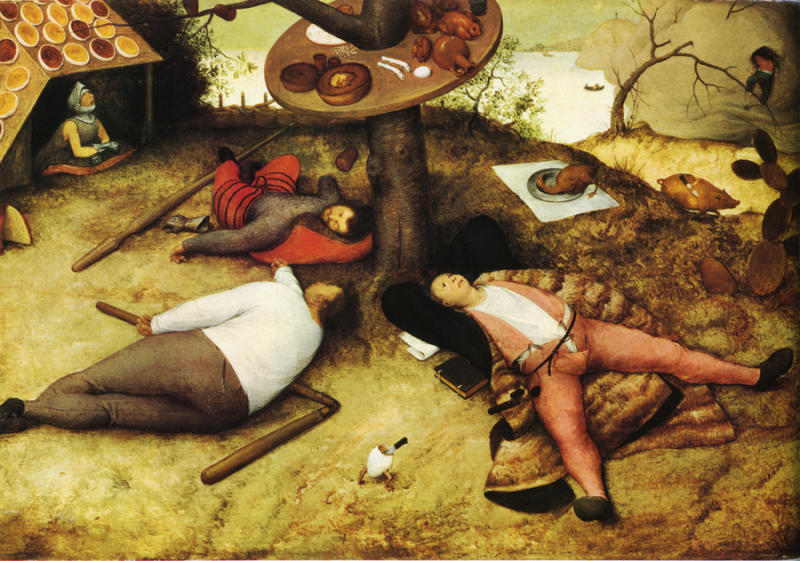
The Land of Cockaigne, by Pieter Brueghel the Elder

Leptin promotes inflammation. The hormone provides an interesting link between obesity and pathophysiological processes such as insulin resistance and atherosclerosis, and disorders such as autoimmune and cardiovascular diseases and the metabolic syndrome. Increased serum leptin levels in obesity and metabolic syndrome support the view that these disorders are in fact low-grade systemic inflammatory diseases, characterized by increased concentrations of proinflammatory cytokines like interleukin-6, tumor necrosis factor-α and leptin. Leptin's proinflammatory role suggests that it may link energy homeostasis to the immune system [4,5].Ghrelin is a peptide hormone that stimulates appetite, fat production, and body growth—leading to increased food intake and body weight. It is secreted into the circulation from the stomach, but is also synthesised in a number of other tissues, including the kidney, pituitary, and hypothalamus, suggesting that the hormone has both distant and local (endocrine and paracrine) effects. These effects include stimulating the secretion of growth hormone, prolactin, and adrenocorticotropic hormone, and a diabetogenic effect on carbohydrate metabolism [6].

## The New Study

In this study of 1,024 participants in the population-based Wisconsin Sleep Cohort Study [7], Mignot and colleagues found that in persons sleeping less than 8 hours, increased BMI was proportional to decreased sleep [3]. The researchers also found that shorter sleep times were associated with increased circulating ghrelin and decreased leptin, a hormonal pattern that is consistent with decreased energy expenditure and increased appetite and obesity.

These findings confirm earlier clinical reports on the effects of sleep deprivation and extend them to include naturalistic sleep in a large, community-based population. The study provides an exciting addition to the growing literature showing relationships between sleep curtailment, metabolic hormones, and metabolic disorders (including obesity). The data have important implications for our understanding of obesity and related disorders in the general population, with one caveat: the study population was enriched with snorers, making the results less applicable to a general population.

Mignot and colleagues' data are in accord with human and animal studies that show that experimental curtailment of sleep leads to lower levels of leptin [8,9,10,11] and increased ghrelin [12]. The new study therefore lends some support to the interpretation that reduced sleep levels cause the hormonal changes.

But there is also evidence of opposite effects—that is, that administration of leptin [13] and ghrelin can alter sleep. Ghrelin administration has been found to increase non-REM sleep in humans and mice, possibly via its interactions with the sleep-inducing peptide growth hormone releasing hormone (GHRH). Ghrelin is an endogenous ligand of the growth hormone secretagogue receptor, making it a candidate for an endogenous sleep-promoting factor [14]. Mignot and colleagues' study is congruent with the idea that inadequate sleep enhances ghrelin secretion, which in turn acts as an endogenous sleep factor in humans. This is an important new area of research that could conceivably lead to more physiological sleep aids than are currently available, with profound implications for improved public health.

Overall, the available studies suggest the presence of reciprocal interactions between metabolic hormones and sleep, relationships that are poorly understood at present. Does sleep interact with metabolic hormones directly or via intervening factors such as sleep-related breathing disorders? Patients with obstructive sleep apnea have impaired sleep and higher ghrelin levels than BMI-matched controls, and treatment with continuous positive airway pressure reduces ghrelin to control levels [15]. Although sleep-disordered breathing (SDB) was measured in the present study, the SDB analyses were not shown, making it difficult to evaluate the influence of SDB on ghrelin and leptin in this population.

There is a clear need for well-controlled, population-based studies that allow us to examine multiple relevant factors simultaneously. The present study highlights the importance of shortened sleep in relation to obesity, leptin, and ghrelin, a good start toward this goal.

## Sleep and Public Health

Many other important questions remain, such as the roles that other hormones, cytokines, and SDB play in obesity. Many of the unanswered questions have important implications for public health. For example, diabetes, visceral obesity, hypertension, and hyperinsulinemia commonly aggregate together in large populations, and are considered a “metabolic syndrome” that has been linked to SDB [16] and to inflammatory disorders [17]. To what extent does long-term sleep curtailment contribute to these and related public health issues?

The possible role of sleep restriction in autoimmune and inflammatory disorders is of particular interest in light of recent findings linking immune function with ghrelin and leptin. Ghrelin and its receptor are expressed in human T-lymphocytes, where they can inhibit cytokine activation, including interleukins, tumor necrosis factor-α and leptin [18]. Conversely, leptin stimulates cytokine activation and immune-cell proliferation, an effect that predisposes to inflammatory conditions [4]. Is it possible, then, that sleep-related changes in leptin and ghrelin influence the development of metabolic and immune disorders? Can biologically restorative sleep reverse disease progression? Can biologically restorative sleep be defined on the basis of metabolic hormone responses? Future research may answer some of these and other questions, further elucidating the role of sleep in public health.

## Abbreviations

BMI: body mass index
SDB: sleep-disordered breathing
